# Integrated Evaluation of the Multifunctional DPP-IV and ACE Inhibitory Effect of Soybean and Pea Protein Hydrolysates

**DOI:** 10.3390/nu14122379

**Published:** 2022-06-08

**Authors:** Carlotta Bollati, Ruoxian Xu, Giovanna Boschin, Martina Bartolomei, Fabrizio Rivardo, Jianqiang Li, Anna Arnoldi, Carmen Lammi

**Affiliations:** 1 Department of Pharmaceutical Sciences, University of Milan, Via Mangiagalli 25, 20133 Milan, Italy; carlotta.bollati@unimi.it (C.B.); ruoxian.xu@unimi.it (R.X.); giovanna.boschin@unimi.it (G.B.); martina.bartolomei@unimi.it (M.B.); jianqiang.li@unimi.it (J.L.); anna.arnoldi@unimi.it (A.A.); 2 A. Costantino & C. Spa, Via Francesco Romana 11, 10083 Torino, Italy; frivardo@acostantino.com

**Keywords:** ACE1, bioactive peptides, diabetes, DPP-IV, protein hydrolysates, hypertension

## Abstract

Nowadays, notwithstanding their nutritional and technological properties, food bioactive peptides from plant sources garner increasing attention for their ability to impart more than one beneficial effect on human health. Legumes, which stand out thanks to their high protein content, represent valuable sources of bioactive peptides. In this context, this study focused on the characterization of the potential pleotropic activity of two commercially available soybean (SH) and pea (PH) protein hydrolysates, respectively. Since the biological activity of a specific protein hydrolysate is strictly correlated with its chemical composition, the first aim of the study was to identify the compositions of the SH and PH peptides. Peptidomic analysis revealed that most of the identified peptides within both mixtures belong to storage proteins. Interestingly, according to the BIOPEP-UWM database, all the peptides contain more than one active motive with known inhibitory angiotensin converting enzyme (ACE) and dipeptidyl-dipeptidases (DPP)-IV sequences. Indeed, the results indicated that both SH and PH inhibit DPP-IV and ACE activity with a dose-response trend and IC_50_ values equal to 1.15 ± 0.004 and 1.33 ± 0.004 mg/mL, and 0.33 ± 0.01 and 0.61 ± 0.05 mg/mL, respectively. In addition, both hydrolysates reduced the activity of DPP-IV and ACE enzymes which are expressed on the surface of human intestinal Caco-2 cells. These findings clearly support that notion that SH and PH may represent new ingredients with anti-diabetic and hypotensive effects for the development of innovative multifunctional foods and/or nutraceuticals for the prevention of metabolic syndrome.

## 1. Introduction

Food bioactive peptides are short protein fragments (2–20 amino acid residues in length) that, in addition to their known nutritional value, are able to modulate physiological pathways, thereby exerting a positive impact on human health [1]. Hence, both animal and plant foods or by-products with high protein content represent valuable sources of functional bioactive peptides, which can be produced either by enzymatic hydrolysis (using proteolytic enzymes from either plants or microbes), hydrolysis with digestive enzymes (simulated gastrointestinal digestion), or by fermentation. Some studies also used a combination of these methods to produce peptides with a biological activity [2].

Legumes, pseudocereals, and hempseed are among the plant foods which can be considered good sources of bioactive peptides [3,4,5,6].

In this panorama, legumes, which stand out thanks to their high protein content, are a cheap, sustainable, and a healthy source of nutrition. Notably, soybeans (*Glycine max*) are on average composed of ~35–40% protein [7]. Clinical studies have linked the consumption of soy-based food with a reduced risk of developing a number of chronic diseases, such as obesity, hypercholesterolemia, and insulin-resistance/type II diabetes. As for the active substance in soy foods, protein plays a role in cardiovascular disease prevention [3,8], and some cholesterol-lowering and anti-diabetic peptides have already been singled out in glycinin and β-conglycinin sequences, respectively [9].

In addition to soybean, pea (*Pisum sativum* L.) represents one of the major legumes in the world and it is composed of ~26% protein [7]. Thanks to its excellent yields, availability, and its low production costs, pea is most widely used as a source of commercial proteins for different purpose [10]. Many studies have highlighted the health benefits associated with the consumption of pea protein. In particular, pea protein and its hydrolysates exert antioxidant, antihypertensive, and hypocholesterolemic activities [11,12,13,14].

In general, most previous studies in this area have focused on the consumption of pea and soybean proteins, but the health-promoting activity does not lie in the protein but in the peptides, which are encrypted within the protein and released upon digestion/hydrolysis. Hence, with a more mature perception of the phenomenon, owing to the presence of numerous bioactive peptides, these protein hydrolysates may provide more than one biological activity, therefore eliciting multiple health benefits [15,16,17,18]. For this reason, the production of hydrolysates with multifunctional behavior represents a valid strategy in the development of new generations of functional foods and nutraceuticals [19].

Indeed, this study focused on the deep characterization of the potential pleotropic health-promoting behavior of two commercially available soybean (SH) and pea (PH) protein hydrolysates, respectively. Both SH and PH are obtained by industrial processes, beginning with the selection of soybean and pea protein sources (isolated proteins or enriched protein flours) in combination with a hydrolysis process which is able to deliver an optimized performance in term of structure and, thanks to the resulting taste being almost neutral, allows for a nutritional contribution to food and beverage formulations. SH and PH are then filtered and concentrated before spray drying to obtain a purified product for different food applications.

In addition to, and in part thanks to, their nutritional and technological properties (as a smoothing and whipping agent), SH and PH are successfully used in every formulation to replace nutritional content of animal origin, and they may also be exploited as new valuable and pleotropic ingredients for making innovative multifunctional foods and/or nutraceuticals.

Thus, since the biological activity of a specific protein hydrolysate is strictly correlated with its chemical composition, the first aim of this study was to identify the compositions of SH and PH peptides by peptidomic analysis. Then, their potential hypotensive and anti-diabetic activities were evaluated by initially measuring the ability of both hydrolysates to inhibit, in vitro, angiotensin converting enzyme (ACE) and dipeptidyl-dipeptidases (DPP)-IV activity and, afterwards, by carrying out experiments using Caco-2 cells that express both enzymes on their membrane surfaces.

## 2. Materials and Methods

### 2.1. Chemicals

All the commercial chemicals which were used are listed in the Supplementary Materials.

#### SH and PH Samples Description

A. Costantino & C. S.p.a (Italy), supplied the soybean and pea hydrolysates as spray dried samples (Soy Peptone FM batch 221/00351, 100 g; Pea Protein Hydrolysate GT Plus batch 221F0001, 100 g) directly from the production process. See the Supplementary Materials for the Technical data sheet of both samples.

### 2.2. SH and PH Ultrafiltration

Before proceeding to the assessment of biological activity, SH and PH were passed through ultrafiltration (UF) membranes with a 3 kDa cut-off, using a Millipore UF system (Millipore, Bedford, MA, USA). The recovered peptides solutions (SH (F3) and PH (F3)) were lyophilized and stored −80 °C until use.

### 2.3. Mass Spectrometry Analysis (HPLC Chip ESI-MS/MS)

SH and PH samples were analyzed by HPLC CHIP-ESI-MS/MS. All further details and experimental conditions are described in the Supplementary Materials.

### 2.4. Biochemical Investigation of DPP-IV and ACE Inhibitory Activity of SH and PH Peptides

#### 2.4.1. In Vitro DPP-IV Activity Assay

The experiments were carried out in a half-volume 96-well solid plate (white) with SH and PH at final concentration range of 0.01–2.0 mg/mL and using conditions previously optimized [20]. For further details, see the Supplementary Materials.

#### 2.4.2. In Vitro Measurement of ACE Inhibitory Activity

In order to assess their ACE-inhibitory activity, the SH and PH hydrolysates were tested as previously reported [21,22]. The detailed procedures are available in the Supplementary Materials.

### 2.5. Cellular Measurement of SH and PH Inhibitory Effect of DPP-IV and ACE Activities

#### 2.5.1. Cell Culture Conditions

Caco-2 cells, obtained from INSERM (Paris, France), were routinely sub-cultured following conditions which have been already optimized [16] and detailed reported in the Supplementary Materials.

#### 2.5.2. Evaluation of Caco-2 Cell Viability by MTT Experiments

In order to assess the safe range of concentrations of the SH and PH hydrolysates on Caco-2 cells, preliminary cell viability experiments were carried out using MTT assay. The detailed procedure is shown in the Supplementary Materials.

#### 2.5.3. Evaluation of the Inhibitory Effect of SH and PH on Cellular DPP-IV Activity

A total of 5 × 10^4^ Caco-2 cells/well were seeded in black 96-well plates with a clear bottom. The second day after seeding, the spent medium was discarded and cells were treated with 1.0, 2.5, and 5.0 mg/mL of SH and PH for 1, 3, and 6 h at 37 °C. Experiments were carried out following previously optimized conditions [23]. More details are available in the Supplementary Materials.

#### 2.5.4. Evaluation of the Inhibitory Effect of SH and PH on Cellular ACE1 Activity

Caco-2 cells were seeded on 96-well plates at a density of 5 × 10^4^ cells/well and treated with SH and PH (from 0.1 to 5.0 mg/mL) or vehicle in growth medium for 6 h at 37 °C. The ACE inhibitory activity was measured using the ACE1 Activity Assay Kit (Biovision, Milpitas Blvd., Milpitas, CA, USA). The experimental method is detailed in the Supplementary Materials.

### 2.6. Statistical Analysis

All the data sets were checked for normal distribution by the D’Agostino and Pearson test. Since they are all normally disturbed with *p*-values < 0.05, statistical analyses were carried out by one-way ANOVA (Graphpad Prism 9, GraphPad Software, La Jolla, CA, USA) followed by Tukey’s multiple comparison test. For each assay, at least four independent experiments were performed, and each experiment were performed in triplicate. Values were expressed as means ± SD; *p*-values < 0.05 were considered to be significant.

## 3. Results

### 3.1. Peptidomic Characterization of SH and PH

**Figure 1:**
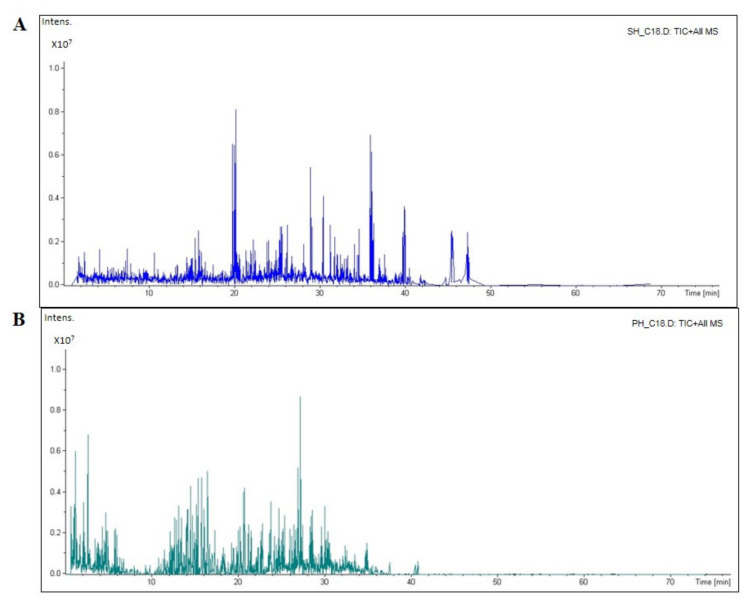
The total ion chromatogram (TIC) of the soybean (SH, (**A**)) and pea (PH, (**B**)) hydrolysates, respectively. The mass spectrometer ran for 70 min. Most of the SH sample peptides were eluted between 20 and 40 min, while the PH peptides were mostly eluted in 30–40 min.

The compositions of the peptides of SH and PH hydrolysates were analyzed by HPLC-ESI-MS/MS. Figure 1 reports the total ion current (TIC) of the MS/MS of eluted peptides, while Table 1 shows the peptide molecular weight distribution of each sample, and the overall peptides, which were identified, are reported in Table S1.

Table 2 lists the identified peptides from the most abundant proteins. Among these, six and nine peptides were identified in the SH and PH samples, respectively. The length of those peptides ranged from 9 to 25 amino acids with a molecular weight in the range 1055 –2422.3 KDa, and in both samples, most of the identified peptides belong to storage proteins. Indeed, 50% of the identified soybean peptides belong to Glycinin G1, whereas in the case of the pea derived peptides, 55% and 33% belong to the Vicilin and Legumin A2 proteins, respectively. Interestingly, according to BIOPEP-UWM database (https://biochemia.uwm.edu.pl/biopep-uwm/, accessed on 20 May 2022), all the peptides contain more than one active motive with both known inhibitory ACE and DPP-IV sequences.

### 3.2. SH and PH Peptides: Biochemical Investigation of DPP-IV and ACE Inhibitory Activities

#### 3.2.1. SH and PH Inhibit In Vitro DPP-IV Activity

**Figure 2:**
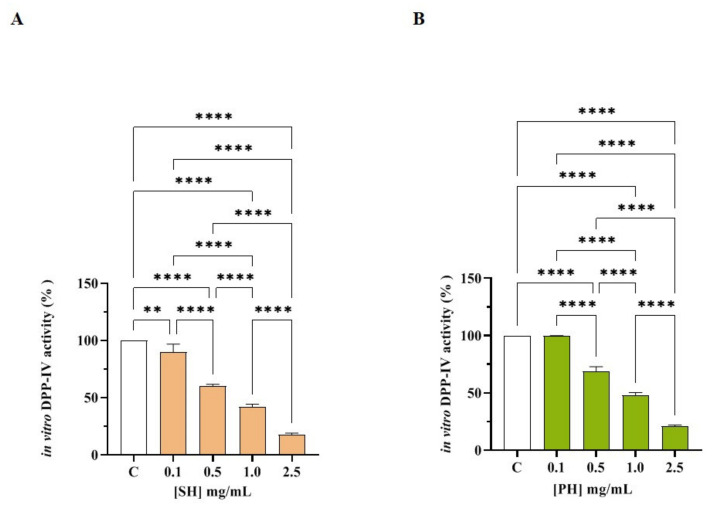
Evaluation of the in vitro inhibitory effects of SH (**A**) and PH (**B**) hydrolysates on human recombinant DPP-IV. Bars represent the average ± SD of three independent experiments in duplicates. **** *p* < 0.0001, ** *p* < 0.01, versus control (C) sample (activity), non-significant (ns) is not shown.

In order to assess the ability of SH and PH to modulate DPP-IV activity, preliminary in vitro experiments were performed using the purified recombinant DPP-IV enzyme. Figure 2A shows that SH drops in vitro DPP-IV activity by 10.18 ± 7.0%, 40.0 ± 1.6%, 58.22 ± 2.57%, and 82.48 ± 1.41% at 0.1, 0.5, 1.0, and 2.5 mg/mL, respectively. Figure 2B indicates that PH decreases DPP-IV activity in vitro by 0.11 ± 0.21%, 31.0 ± 3.9%, 52.05 ± 2.31%, and 79.1 ± 1.04% at 0.1, 0.5, 1.0, and 2.5 mg/mL, respectively. Both SH and PH inhibit the enzyme with a dose-response trend and calculated IC_50_ values equal to 1.15 ± 0.004 and 1.33 ± 0.004, respectively.

In parallel, the in vitro DPP-IV activity inhibition of both hydrolysates was confirmed by the experiments conducted on the low molecular weight fractions (<3 kDa) of SH (F3) and PH (F3), as reported in Figure S1. Notably, SH (F3) inhibits DPP-IV activity in vitro by 12.7 ± 4.9%, 23.9 ± 4.56%, 41.9 ± 2.56%, 57.3 ± 2.3%, and 79.5 ± 2.5% at 0.01, 0.1, 0.5, 1.0, and 2.0 mg/mL, respectively (Figure S1A), and PH (S3) reduced the enzymatic activity by 8.5 ± 2.01%, 15.1 ± 4.21%, 41.6 ± 3.4%, 56.8 ± 4.4%, and 79.1 ± 1.70% at the same concentrations (Figure S1B). Indeed, both SH (F3) and PH (F3) decreased DPP-IV activity with a dose-response trend and IC_50_ values equal to 0.82 ± 0.01 and 1.0 ± 0.01 mg/mL, respectively. Comparing the IC_50_ values obtained by analyzing the total SH and PH hydrolysates with the those obtained by testing the <3 kDa fractions of SH and PH, it is evident that the short peptides which are contained within SH and PH hydrolysates, respectively, are those responsible of DPP-IV inhibitory activity.

#### 3.2.2. SH and PH Peptides Inhibit In Vitro ACE Activity

**Figure 3:**
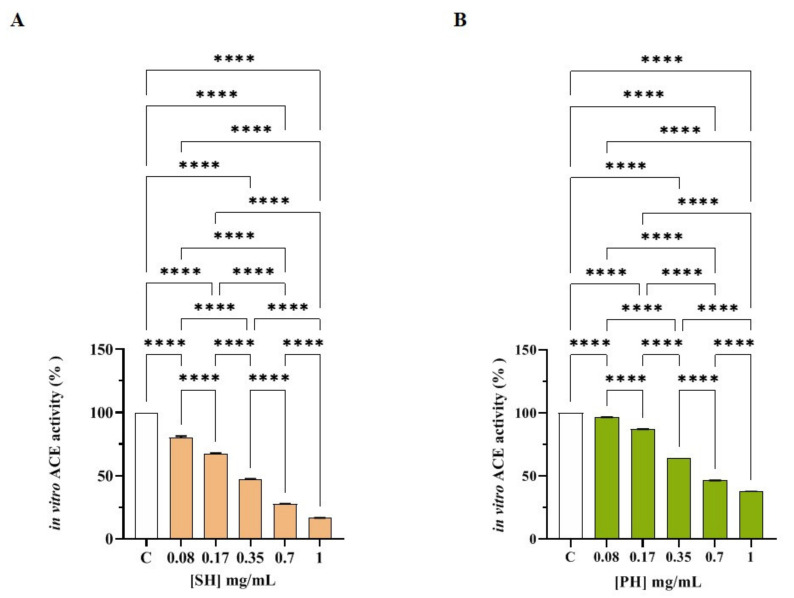
Assessment of the in vitro ACE-inhibitory effects of SH (**A**) and PH (**B**) hydrolysates. Bars represent the sd of three independent experiments in duplicate. **** *p* < 0.0001 versus control sample (C).

Both SH and PH efficiently inhibited ACE activity with a dose-response trend and calculated IC_50_ values that were 0.33± 0.01 and 0.61 ± 0.05 mg/mL, respectively (Figure 3A,B). More specifically, SH inhibited ACE activity by 19.88 ± 1.21%, 32.74 ± 0.57%, 53.01 ± 0.43%, 72.35 ± 0.16%, and 83.38 ± 0.06% at 0.08, 0.17, 0.35, 0.7, and 1.0 mg/mL, respectively (Figure 2A), whereas PH reduced ACE activity by 3.55 ± 0.07%, 12.92 ± 0.11%, 35.40 ± 0.01%, 53.51 ± 0.11%, and 62.23 ± 0.15% at the same concentrations (Figure 3B).

In parallel, the in vitro ACE activity inhibition of both hydrolysates was confirmed by the experiments conducted on the low molecular weight fractions (<3 kDa) of both SH (F3) and PH (F3), as shown in Figure S2. In particular, SH (F3) inhibits ACE activity in vitro by 9.15 ± 0.07%, 24.62 ± 0.13%, 48.39 ± 0.02%, 63.29 ± 0.02%, and 74.09 ± 0.08% at 0.08, 0.17, 0.35, 0.7, and 1.0 mg/mL, respectively (Figure S2A), whereas PH (F3) reduced the enzymatic activity by 17.21 ± 0.10%, 26.74 ± 0.25%, 44.26 ± 0.02%, 63.96 ± 0.01%, and 74.64 ± 0.08% at the same concentrations (Figure S2B).

Indeed, both SH (F3) and PH (F3) reduced the ACE activity with a dose-response trend and IC_50_ values equal to 0.40 ± 0.01 and 0.43 ± 0.01 mg/mL, respectively, clearly suggesting that the short peptides which are contained within SH and PH hydrolysates are those responsible for ACE inhibitory activity.

Finally, Table 3 summarizes the comparison of IC_50_ values of both samples against DPP-IV and ACE.

### 3.3. Cellular Assessment of DPP-IV and ACE Inhibition by SH and PH Peptides

#### 3.3.1. Effect of SH and PH Peptides on Caco-2 Cell Viability

**Figure 4:**
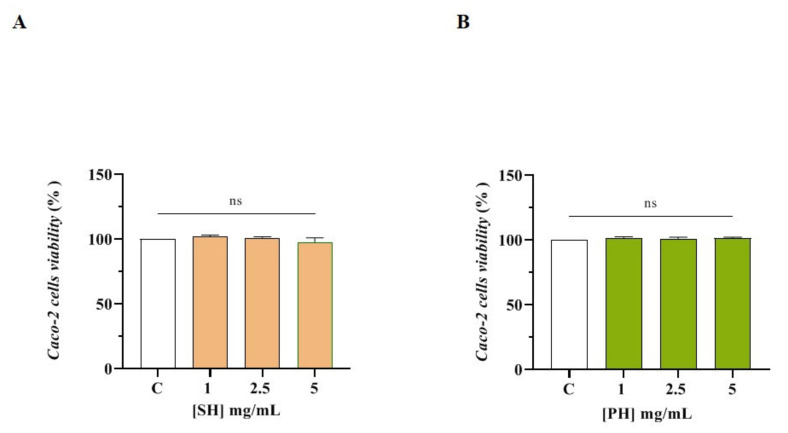
MTT assay. Effect of SH (**A**) and PH (**B**) hydrolysates on Caco-2 cells viability. Data represent the averages ± SD of four independent experiments performed in triplicate.

The MTT assay was used for assessing the safe range of concentrations of the SH and PH hydrolysates on Caco-2 cells. After a 48 h treatment, any effect on the Caco-2 cell viability was observed in the range of 1.0–5.0 mg/mL versus untreated cells (C) (Figure 4).

**Figure 5:**
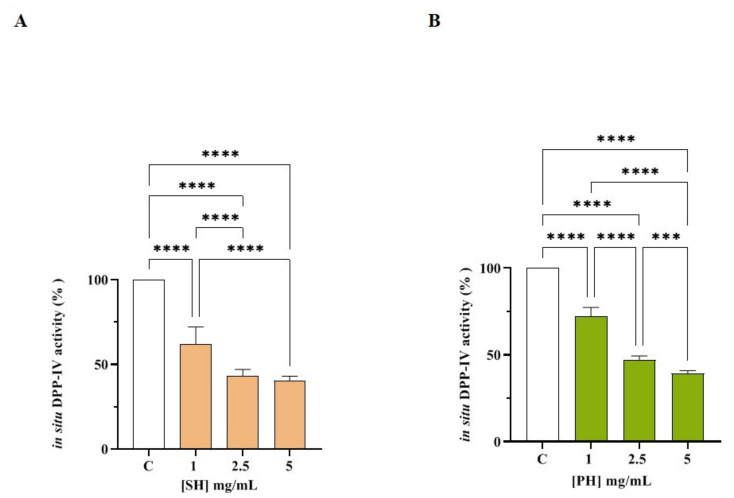
Effect of SH (**A**) and PH (**B**) on the cellular DPP-IV activity. The data points represent the averages ± SD of four independent experiments performed in triplicate. All data sets were analyzed by one-way ANOVA followed by Tukey’s post-hoc test; C: control sample (H_2_O), **** *p* < 0.0001, *** *p* < 0.001, non-significant (ns) is not shown.

The ability of SH and PH to inhibit DPP-IV was then investigated in cell-based conditions using Caco-2 cells, which express high levels of this protease on their membranes. Briefly, these cells were treated with SH and PH (1.0- 5.0 mg/mL) and their DPP-IV inhibitory effects were assessed in a kinetic mode after 1, 3, and 6 h (Figure S3A,C). Figure S3A shows that, after 1 h, SH inhibited cellular DPP-IV activity by 25.07 ± 8.07%, 36.91 ± 2.37%, and 40.32 ± 2.46 at 1.0, 2.5, and 5 mg/mL, respectively, and by 25.07 ± 6.78%, 42.46 ± 6.49%, and 48.69 ± 7.29% at the same concentrations after 3 h. The maximum reductions in DPP-IV activity were observed at 6 h (Figure S3A and Figure 5A), where SH inhibited cellular DPP-IV activity by 37.9 ± 10.0%, 56.6 ± 3.6%, and 59.4 ± 2.4% at 1.0, 2.5, and 5 mg/mL, respectively, versus untreated cells, following a dose-response trend, confirming the in vitro results.

As indicated in Figure S3C, after 1 h, the PH sample inhibited cellular DPP-IV activity by 19.9 ± 8.2%, 40.9 ± 3.6%, and 47.4 ± 3.3% at 1.0, 2.5, and 5 mg/mL, respectively, and by 22.6 ± 5.4%, 47.9 ± 2.79%, and 53.6 ± 2.3% at the same concentrations after 3 h (Figure S3C). The maximum inhibition of DPP-IV activity was observed after 6 h (Figure S3C, Figure 5B), where PH inhibited cellular DPP-IV activity by 27.9 ± 5.3%, 52.8 ± 2.2%, and 60.7 ± 1.6% at 1.0, 2.5 and 5 mg/mL, respectively, versus untreated cells, supporting the in vitro results.

In parallel, the in situ DPP-IV activity inhibition of both hydrolysates was confirmed by the experiments carried out on the low molecular weight fractions (<3 kDa) of both SH (F3) and PH (F3), as shown in Figure S3B,D. In particular, Figure S3B shows that, after 1 h, SH (F3) inhibited cellular DPP-IV activity by 10.4 ± 8.3%, 37.2 ± 4.1%, and 42.3 ± 3.10% at 1.0, 2.5, and 5 mg/mL, respectively, and by 13.62 ± 6.88%, 43.1 ± 5.91%, and 47.9 ± 4.4% at the same concentrations after 3 h. The highest decreases in DPP-IV activity were observed at 6 h (Figure S3B), where SH (F3) inhibited cellular DPP-IV activity by 17.7 ± 4.9%, 54 ± 1.6%, and 61.5 ± 3.9% at 1.0, 2.5, and 5 mg/mL, respectively, versus untreated cells, in a dose-dependent manner, supporting the in vitro results.

Similarly, as shown in Figure S3D, PH (F3) inhibited cellular DPP-IV activity by 6.8 ± 6.5%, 31.6 ± 5.5%, and 45.5 ± 4.9% at 1.0, 2.5, and 5 mg/mL, respectively, after 1 h, and by 7.25 ± 4.03%, 48.67 ± 2.42%, and 54.11 ± 3.09% at the same concentrations after 3 h. The highest inhibition of DPP-IV activity was observed at 6 h (Figure S3D), where PH (F3) inhibited cellular enzymatic activity by 14.4 ± 3.9%, 60.2 ± 1.5%, and 61.8 ± 2.7% at 1.0, 2.5, and 5 mg/mL, respectively, versus untreated cells, confirming the in vitro results.

#### 3.3.2. SH and PH Inhibit ACE Activity Expressed on Human Intestinal Caco-2 Cells

**Figure 6:**
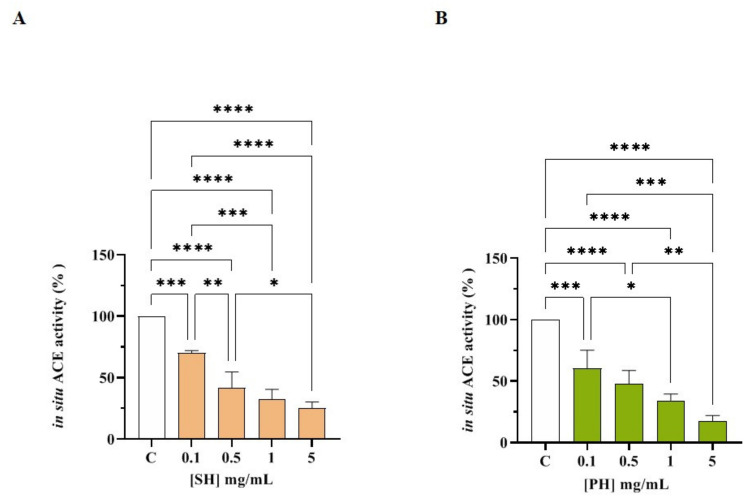
ACE inhibitory effects of SH (**A**) and PH (**B**) hydrolysates in cell-based conditions. Bars represent the SD of three independent experiments in triplicate. **** *p* < 0.0001, *** *p* < 0.001, ** *p* < 0.01, * *p* < 0.05 versus control sample (C), non-significant (ns) is not shown.

Human intestinal Caco-2 cells were treated with SH and PH (1.0–5.0 mg/mL) for 6 h. The ACE activity was measured in the presence of a fluorescent substrate using cell lysates. The results indicated that both hydrolysates reduced cellular ACE activity with a dose-response trend. In more detail, SH reduced the enzyme activity by 29.9 ± 1.8%, 57.9 ± 12.6%, 67.5 ± 8.1%, and 74.6 ± 4.9% at 0.1, 0.5, 1.0, and 5.0 mg/mL, respectively (Figure 6A), whereas PH hydrolysate reduced it by 39.6 ± 14.6%, 52.2 ± 10.6%, 64.7 ± 5.2, and 82.3 ± 4.2%, respectively, at the same concentrations (Figure 6B). In parallel, the cellular ACE activity inhibition of both hydrolysates was confirmed by the experiments performed on the low molecular fractions (3 kDa) of both SH and PH (Figure S4). Notably, SH (F3) inhibited cellular ACE activity by 10.1 ± 4.5%, 47.6 ± 2.7%, 61.0 ± 7.5%, and 76.2 ± 2.3% at 0.1, 0.5, 1.0, and 5.0 mg/mL, respectively (Figure S4A), whereas PH (F3) reduced the cellular enzymatic activity by 32.0 ± 13.7%, 42.7 ± 11.3%, 60.5 ± 13.5%, and 69.5 ± 11.3% at the same concentrations (Figure S4B).

## 4. Discussion

In recent decades, many studies have clearly demonstrated the ability of food protein hydrolysates to modulate ACE or DPP-IV activity [20,24,25]. In particular, hydrophobic medium-length and/or shorter peptides from different food sources are considered to be mostly responsible for the inhibition of both enzymes [24,25]. In this panorama, the main limitation of most of previous studies lies in the fact that the characterization of the DPP-IV or ACE inhibitory property of food hydrolysates was performed by analyzing each bioactivity without taking into account that the same hydrolysate may be endowed with both activities. In addition, most works available in the literature rely exclusively on in vitro tests for assessing the biological activity: this is particularly true in the case of the inhibition of DPP-IV and ACE activity. In light of these observations, a standout feature of this study is that the multifunctional DPP-IV and ACE inhibitory activities of two commercially available soybean (SH) and pea (PH) protein hydrolysates were evaluated together using in vitro tests which were implemented by cellular assays that permitted deeper insights into the mechanism of action and, contextually, a consideration of other relevant issues, such as metabolism (this is particularly true when Caco-2 cells were employed).

Overall, the present study demonstrates that SH and PH are effective at reducing DPP-IV and ACE activity in both cell-free and cell-based conditions. More specifically, as indicated in the Table 3, when comparing the calculated IC_50_ values, it is clear that SH and PH display the same ability to reduce DPP-IV activity, with SH being about 2-fold more potent than PH in terms of ACE inhibition (***, *p* < 0.001). In addition, the results indicated that SH and PH are 3- and 2-fold more potent as ACE than DPP-IV inhibitors, respectively (****, *p* < 0.0001). Moreover, comparing the IC_50_ values of each total hydrolysate with those corresponding to each low molecular weight fraction (<3 kDa) (Table 3), it is clear that the medium-length and shorter peptides, which are abundant in each total hydrolysate (Table 1), are responsible for the biological activities, even though, in the case of SH and SH (F3), the same IC_50_ values were observed against ACE enzyme. Interestingly, Table 2 indicates that all the most abundant peptides identified within both SH and PH contained at least one motive with DPP-IV and ACE inhibitory activity that has already been demonstrated, explaining why these hydrolysates are active on two different targets, such as ACE and DPP-IV.

As reported in the Table 3, in the case of PH, the low molecular weight fractions (<3 kDa) are about 0.8- and 1,5-fold more potent than total PH as DPP-IV and ACE inhibitors, respectively (****, *p* < 0.0001). This result might be explained considering that the bioactivity of total hydrolysate depends strictly on its total composition, including the inactive and active species and possible synergistic or antagonist effects [26,27]. Therefore, it is reasonable to conclude that longer peptides of PH (57.2%) may affect the activity exerted by the medium-length and shorter ones (42.8%) when present in PH hydrolysate. A similar trend was observed for SH. In fact, the SH (F3) is more active than the total hydrolysate against DPP-IV (***, *p* < 0.001), whereas a similar IC_50_ value against the ACE target was calculated (Table 3).

Recently, it was demonstrated that soybean hydrolysates obtained using pepsin and trypsin reduced in vitro DPP-IV activity by 16.3% and 31.4%, and by 15.3% and 11.0%, respectively, at 1.0 and 2.5 mg/mL [18]. Other recent studies demonstrated that the protein hydrolysates from germinated and non-germinated soybean, obtained after simulated gastrointestinal digestion, show a modest ability to inhibit the DPP-IV [28,29]. In addition, soybean hydrolysates obtained using Corolase L10, Promod 144 MG, or Protamex reduced the enzyme, with IC_50_ values of 2.5, 0.86, and 0.96 mg/mL, respectively [28]. Indeed, SH is more active than hydrolysates obtained using pepsin, trypsin, and Corolase, whereas its activity is very similar to those of peptide mixtures obtained using both Promod and Protamex.

Pea proteins digested with Corolase L10 and Promod 144 MG inhibited DPP-IV activity with IC_50_ values higher than 2.5 mg/mL, whereas the pea hydrolysate obtained using Protamex dropped the enzyme activity with an IC_50_ value of 0.96 mg/mL [28]. In this case, PH displays a DPP-IV activity totally in line with those of hydrolysates obtained using Protamex, whereas it is more active than the peptide mixtures obtained using Corolase and Promod.

Soybean proteins extracted with microwave-assisted technology and hydrolysate using Alcalase inhibited the ACE enzyme, and peptides belonging to the low molecular weight fraction were responsible for the biological activities. Moreover, it was also demonstrated that pea proteins digested using a thermolysin-generated peptide mixture exerted ACE inhibitory properties in a spontaneous hypertensive rat (SHR) model and in a clinical study [12,14].

Interestingly, among the peptides belonging to the low molecular weight fraction (<3 kDa) of pea hydrolysate, peptide LTFPG was isolated, whose hypotensive activity has been demonstrated both in vitro and in vivo in the SHR model [12]. Notably, LTFPG is a conserved active peptide that has also been identified and characterized in a lupin sample [15].

DPP-IV and ACE are important membrane peptidases which are physiological expressed by many tissues; i.e., intestine [30,31]. Indeed, human intestinal Caco-2 cells represent a reliable model which has been already developed and validated for the study of peptides with DPP-IV or ACE inhibitory properties [23,32,33]. In this study, it was clearly demonstrated that both SH and PH maintain their ability to reduce the activity of both DPP-IV and ACE on Caco-2 cells, even though both hydrolysates are active at a concentration ranging between 0.1 and 5 mg/mL, indicating that SH and PH are less active in cell-based than in cell-free conditions, respectively. Similar results have been previously obtained on peptic and tryptic hydrolysates of spirulina and chlorella proteins, respectively [32,34,35]. Also, in those cases, it was observed that all the tested hydrolysates were more active in cell-free than in cell-based assays, respectively. The reduced activity in the cellular assays may be explained considering the metabolic ability of Caco-2 cells [32]. Indeed, the intestinal brush border expresses many active proteases and peptidases that might actively hydrolyze food peptides modulating their bioactivity through the production of new breakdown fragments. Therefore, the intestine plays an important role not only in the process of valuable nutrient absorption, but also in actively modulating the physico-chemical and biological profiles of food protein hydrolysates. All these biochemical and cellular results represent an important starting point for future investigations of SH and PH through in vivo and clinical studies on suitable animal models and human volunteers in order to obtain a “proof of concept” with respect to their multifunctional and pleotropic behavior.

## 5. Conclusions

In conclusion, our results indicate that SH and PH are multifunctional hydrolysates endowed with both anti-diabetic and hypotensive activity. It is doubtless that they are among the most potent DPP-IV and ACE inhibitor hydrolysates reported in the literature, suggesting that they may be successfully used as new valuable ingredients for the development of innovative functional foods and or dietary supplements for the prevention of cardiovascular disease and metabolic syndrome. Therefore, future in vivo and clinical studies need to be undertaken for their benefits to be better exploited in the nutraceutical sector.

## Figures and Tables

**Table 1:** Molecular weight distribution of SH and PH peptides.

Hydrolysate	MW > 3 kDa (%)	MW < 3 kDa (%)
SH	53.6	46.4
PH	57.15	42.85

**Table 2:** SH and PH peptides from the most abundant protein, with ACE and DPP-IV inhibitory activity.

Hydrolysate	Protein Name	Peptide Sequence	Intensity	ACE Inhibitor Sequence ^a^	DPP-IV Inhibitor Sequence ^a^
SH	Ankyrin repeat domain-containing protein 52	IRSWIVQVMS	5.11 × 10^7^	IVQ, VQV, VM	WI, IR, QV, SW, VM, VQ
	Glycinin G1	VSIIDTNSLENQLDQ	4.56 × 10^7^		SL, DQ, II, NQ, QL, SI, TN, VS
		IIDTNSLENQLDQMPR	2.07 × 10^7^	PR	MP, SL, DQ, II, NQ, QL TN
		ANSLLNALPEEVIQ	1.75 × 10^7^	EV, LN, ALP, LP	LP, LL, AL, SL, EV, IQ, LN, NA, VI
	Hydrolase_4 domain-containing protein	AAEGGGFSDPAPAPPRLAIPEV	1.45 × 10^7^	PR, AIP, IP, AP, LA, AA, GF, GG, AI, EG, PAP, EV, PP	PP, LA, AP PA, IP, AA AE, DP, EG EV, GF, GG, RL
	DNA-directed RNA polymerase (fragment)	FDIYRVMRPGEPPTMDSAEAMFNA	1.48 × 10^7^	IY, MF, GEP, RP, GE, EA, PG, PT, PP, VM	PP, RP, EP, AE, FN, GE, MF, MR, NA, PG, PT, TM, VM, YR
PH	Vicilin 47k	EITPEKNQQLQDLDIFVN	2.26 × 10^7^	IF, EI, LQ EK, TP	TP, EK, EI NQ, QD, QL, QQ, VN
		NQQLQDLDIFVN	2.80 × 10^7^	IF, LQ	NQ, QD, QL, QQ, VN
		KNQQLQDLDIFVN	7.09 × 10^7^	IF, LQ	NQ, QD, QL, QQ VN
	Vicilin	ITPEKNPQLQDLDIFVN	1.58 × 10^7^	IF, LQ PQ, EK, TP	TP, NP, EK PQ, QD, QL, VN
		KNPQLQDLDIFVN	5.13 × 10^7^	IF, LQ PQ	NP, PQ, QD QL, VN
	AsmA family protein	GGLSFDRKAAKTTASGGLTLSKADA	2.73 × 10^7^	AA, GL, DA, GG, SG, SF, KA, TLS, DR	KA, TA, TT GL, AA, AD AS, DR, GG KT, LT, RK SF, SK, TL
	Legumin A2	LFGQAGLDPLPVDVGA-NGRL	1.80 × 10^7^	PLP, RL LF, PL, VG GA, GL, AG GR, FG, GQ NG, LP	LP, GA, GL PL, AG, DP NG, PV, QA RL, VD, VG
		ALEPDNRIE	1.53× 10^7^	IE, ALEP	EP, AL, DN NR, RI
		SVINNLPLDVVA	4.96 × 10^7^	PL, LPL, LP	VA, LP, VV LPL, PL, IN NL, NN, SV, VI

^a^ According to the BIOPEP-UWM database; https://biochemia.uwm.edu.pl/biopep-uwm/ accessed on 20 May 2022.

**Table 3:** IC_50_ values obtained testing SH, PH and their corresponding low molecular fractions (<3 kDa) against DPP-IV and ACE targets.

	IC_50_ (mg/mL) DPP-IV	IC_50_ (mg/mL) ACE
SH	1.15 ± 0.004	0.33 ± 0.01
PH	1.33 ± 0.004	0.61 ± 0.05
SH <3 kDa (F3)	0.82 ± 0.01	0.40 ± 0.01
PH <3 kDa (F3)	1.0 ± 0.003	0.43 ± 0.01

## Data Availability

Not applicable.

## Supplementary Materials

The following supporting information can be downloaded at: https://www.mdpi.com/article/10.3390/nu14122379/s1, Materials and Methods; Table S1: LC-MS/MS-based identification of SH and PH peptides; Figure S1: Evaluation of the in vitro inhibitory effects of SH (F3) (A) and PH (F3) (B) hydrolysates on human recombinant DPP-IV; Figure S2: Evaluation of the in vitro inhibitory effects of SH (F3) (A) and PH (F3) (B) hydrolysates on ACE; Figure S3: The kinetics of the inhibition of cellular DPP-IV activity after incubating Caco-2 cells with the SH (A), SH (F3) (B), PH (C), and PH (F3) (D) hydrolysates for 1, 3, and 6 h at different concentrations; Figure S4: Evaluation of the inhibitory effects of SH (F3) (A) and PH (F3) (B) hydrolysates on ACE expressed on Caco-2 cell membranes; Technical data sheet S1: technical data sheet of SH hydrolysate; Technical data sheet S2: technical data sheet of PH hydrolysate.

## References

[1] Lammi C., Aiello G., Boschin G., Arnoldi A. (2019). Multifunctional Peptides for the Prevention of Cardiovascular Disease: A New Concept in the Area of Bioactive Food-Derived Peptides. J. Funct. Foods.

[2] Akbarian M., Khani A., Eghbalpour S., Uversky V.N. (2022). Bioactive Peptides: Synthesis, Sources, Applications, and Proposed Mechanisms of Action. Int. J. Mol. Sci..

[3] Fukui K., Tachibana N., Wanezaki S., Tsuzaki S., Takamatsu K., Yamamoto T., Hashimoto Y., Shimoda T. (2002). Isoflavone-Free Soy Protein Prepared by Column Chromatography Reduces Plasma Cholesterol in Rats. J. Agric. Food Chem..

[4] Cruz-Chamorro I., Santos-Sánchez G., Bollati C., Bartolomei M., Li J., Arnoldi A., Lammi C. (2022). Hempseed (Cannabis Sativa) Peptides WVSPLAGRT and IGFLIIWV Exert Anti-Inflammatory Activity in the LPS-Stimulated Human Hepatic Cell Line. J. Agric. Food Chem..

[5] Udenigwe C.C., Aluko R.E. (2012). Food Protein-Derived Bioactive Peptides: Production, Processing, and Potential Health Benefits. J. Food Sci..

[6] Aguchem R.N., Okagu I.U., Okagu O.D., Ndefo J.C., Udenigwe C.C. (2022). A Review on the Techno-Functional, Biological, and Health-Promoting Properties of Hempseed-Derived Proteins and Peptides. J. Food Biochem..

[7] Arnoldi A., Zanoni C., Lammi C., Boschin G. (2015). The Role of Grain Legumes in the Prevention of Hypercholesterolemia and Hypertension. CRC Crit. Rev. Plant Sci..

[8] Liu Z.-M., Ho S.C., Chen Y.-M., Ho S., To K., Tomlinson B., Woo J. (2014). Whole Soy, but Not Purified Daidzein, Had a Favorable Effect on Improvement of Cardiovascular Risks: A 6-Month Randomized, Double-Blind, and Placebo-Controlled Trial in Equol-Producing Postmenopausal Women. Mol. Nutr. Food Res..

[9] Lammi C., Zanoni C., Arnoldi A. (2015). Three Peptides from Soy Glycinin Modulate Glucose Metabolism in Human Hepatic HepG2 Cells. Int. J. Mol. Sci..

[10] Sun X.D., Arntfield S.D. (2011). Gelation Properties of Salt-Extracted Pea Protein Isolate Catalyzed by Microbial Transglutaminase Cross-Linking. Food Hydrocoll..

[11] Ge J., Sun C.X., Corke H., Gul K., Gan R.Y., Fang Y. (2020). The Health Benefits, Functional Properties, Modifications, and Applications of Pea (*Pisum Sativum* L.) Protein: Current Status, Challenges, and Perspectives. Compr. Rev. Food Sci. Food Saf..

[12] Aluko R.E., Girgih A.T., He R., Malomo S., Li H., Offengenden M., Wu J. (2015). Structural and Functional Characterization of Yellow Field Pea Seed (*Pisum Sativum* L.) Protein-Derived Antihypertensive Peptides. Food Res. Int..

[13] Li H., Aluko R.E. (2010). Identification and Inhibitory Properties of Multifunctional Peptides from Pea Protein Hydrolysate. J. Agric. Food Chem..

[14] Li H., Prairie N., Udenigwe C.C., Adebiyi A.P., Tappia P.S., Aukema H.M., Jones P.J.H., Aluko R.E. (2011). Blood Pressure Lowering Effect of a Pea Protein Hydrolysate in Hypertensive Rats and Humans. J. Agric. Food Chem..

[15] Lammi C., Aiello G., Dellafiora L., Bollati C., Boschin G., Ranaldi G., Ferruzza S., Sambuy Y., Galaverna G., Arnoldi A. (2020). Assessment of the Multifunctional Behavior of Lupin Peptide P7 and Its Metabolite Using an Integrated Strategy. J. Agric. Food Chem..

[16] Lammi C., Zanoni C., Ferruzza S., Ranaldi G., Sambuy Y., Arnoldi A. (2016). Hypocholesterolaemic Activity of Lupin Peptides: Investigation on the Crosstalk between Human Enterocytes and Hepatocytes Using a Co-Culture System Including Caco-2 and HepG2 Cells. Nutrients.

[17] Bollati C., Cruz-Chamorro I., Aiello G., Li J., Bartolomei M., Santos-Sánchez G., Ranaldi G., Ferruzza S., Sambuy Y., Arnoldi A. (2022). Investigation of the Intestinal Trans-Epithelial Transport and Antioxidant Activity of Two Hempseed Peptides WVSPLAGRT (H2) and IGFLIIWV (H3). Food Res. Int..

[18] Lammi C., Arnoldi A., Aiello G. (2019). Soybean Peptides Exert Multifunctional Bioactivity Modulating 3-Hydroxy-3-Methylglutaryl-CoA Reductase and Dipeptidyl Peptidase-IV Targets in Vitro. J. Agric. Food Chem..

[19] Chakrabarti S., Guha S., Majumder K. (2018). Food-Derived Bioactive Peptides in Human Health: Challenges and Opportunities. Nutrients.

[20] Lammi C., Zanoni C., Arnoldi A., Vistoli G. (2016). Peptides Derived from Soy and Lupin Protein as Dipeptidyl-Peptidase IV Inhibitors: In Vitro Biochemical Screening and in Silico Molecular Modeling Study. J. Agric. Food Chem..

[21] Boschin G., Scigliuolo G.M., Resta D., Arnoldi A. (2014). Optimization of the Enzymatic Hydrolysis of Lupin (Lupinus) Proteins for Producing ACE-Inhibitory Peptides. J. Agric. Food Chem..

[22] Boschin G., Scigliuolo G.M., Resta D., Arnoldi A. (2014). ACE-Inhibitory Activity of Enzymatic Protein Hydrolysates from Lupin and Other Legumes. Food Chem..

[23] Lammi C., Bollati C., Gelain F., Arnoldi A., Pugliese R. (2019). Enhancement of the Stability and Anti-DPPIV Activity of Hempseed Hydrolysates through Self-Assembling Peptide-Based Hydrogels. Front. Chem..

[24] Daskaya-Dikmen C., Yucetepe A., Karbancioglu-Guler F., Daskaya H., Ozcelik B. (2017). Angiotensin-I-Converting Enzyme (ACE)-Inhibitory Peptides from Plants. Nutrients.

[25] Nongonierma A.B., FitzGerald R.J. (2019). Features of Dipeptidyl Peptidase IV (DPP-IV) Inhibitory Peptides from Dietary Proteins. J. Food Biochem..

[26] Aiello G., Lammi C., Boschin G., Zanoni C., Arnoldi A. (2017). Exploration of Potentially Bioactive Peptides Generated from the Enzymatic Hydrolysis of Hempseed Proteins. J. Agric. Food Chem..

[27] Zanoni C., Aiello G., Arnoldi A., Lammi C. (2017). Hempseed Peptides Exert Hypocholesterolemic Effects with a Statin-Like Mechanism. J. Agric. Food Chem..

[28] Nongonierma A.B., FitzGerald R.J. (2015). Investigation of the Potential of Hemp, Pea, Rice and Soy Protein Hydrolysates as a Source of Dipeptidyl Peptidase IV (DPP-IV) Inhibitory Peptides. Food Dig..

[29] González-Montoya M., Hernández-Ledesma B., Mora-Escobedo R., Martínez-Villaluenga C. (2018). Bioactive Peptides from Germinated Soybean with Anti-Diabetic Potential by Inhibition of Dipeptidyl Peptidase-IV, α-Amylase, and α-Glucosidase Enzymes. Int. J. Mol. Sci..

[30] Howell S., Kenny A.J., Turner A.J. (1992). A Survey of Membrane Peptidases in Two Human Colonic Cell Lines, Caco-2 and HT-29. Biochem. J..

[31] Mentlein R. (2004). Cell-Surface Peptidases. Int. Rev. Cytol..

[32] Li Y., Aiello G., Fassi E.M.A., Boschin G., Bartolomei M., Bollati C., Roda G., Arnoldi A., Grazioso G., Lammi C. (2021). Investigation of Chlorella Pyrenoidosa Protein as a Source of Novel Angiotensin I-Converting Enzyme (Ace) and Dipeptidyl Peptidase-Iv (Dpp-Iv) Inhibitory Peptides. Nutrients.

[33] Lammi C., Boschin G., Bollati C., Arnoldi A., Galaverna G., Dellafiora L. (2021). A Heuristic, Computer-Driven and Top-down Approach to Identify Novel Bioactive Peptides: A Proof-of-Principle on Angiotensin I Converting Enzyme Inhibitory Peptides. Food Res. Int..

[34] Aiello G., Li Y., Boschin G., Bollati C., Arnoldi A., Lammi C. (2019). Chemical and Biological Characterization of Spirulina Protein Hydrolysates: Focus on ACE and DPP-IV Activities Modulation. J. Funct. Foods.

[35] Li Y., Aiello G., Bollati C., Bartolomei M., Arnoldi A., Lammi C. (2020). Phycobiliproteins from Arthrospira Platensis (Spirulina): A New Source of Peptides with Dipeptidyl Peptidase-IV Inhibitory Activity. Nutrients.

