# Supplementary material for: Integrated Evaluation of the Multifunctional DPP-IV and ACE Inhibitory Effect of Soybean and Pea Protein Hydrolysates
Source: Nutrients. 2022 Jun 8;14(12):2379. doi: 10.3390/nu14122379 (PMC9227613; doi:10.3390/nu14122379)
Supplement: Supplementary file 1 [file nutrients-14-02379-s001.zip › nutrients-1758135-supplementary.pdf]

nutrients

MDPI

Integrated evaluation of the multifunctional DPP-IV and ACE inhibitory effect of soybean and pea protein hydrolysates

Carlotta Bollati [1], Ruoxian Xu[1], Giovanna Boschin[1], Martina Bartolomei[1], Fabrizio Rivardo[2], Jianqiang Li[1], Anna Arnoldi[1] and Carmen Lammi[1,*]

[1] Department of Pharmaceutical Sciences, University of Milan, Via Mangiagalli 25, 20133 Milan (Italy); carlotta.bollati@unimi.it (C.B.), ruoxian.xu@unimi.it (R.X.), giovanna.boschin@unimi.it (G.B.), Jianqiang.li@unimi.it (J.L.), anna.arnoldi@unimi.it (A.A.), carmen.lammi@unimi.it (C.L.)

[2] A. Costantino & C. Spa, Via Francesco Romana 11-15 - 10083 Favria (TO) – Italy; frivardo@acostantino.com (F.R.),

* Correspondence: carmen.lammi@unimi.it; Tel.: +39 02/503019372

Table S1: LC-MS/MS based identification of SH and PH peptides.

Hydrolysate.	Protein name	Peptide sequence	Spectrum Intensity
SH	Uncharacterized protein	(W)FNIVGQWAVTT(S)	5.19×10^7
	Ankyrin repeat domain-containing protein 52	(A)IRSWIVQVMS(Q)	5.11×10^7
	Uncharacterized protein	(I)GKQASIIEDPRPGQGKN(L)	2.34×10^7
	Uncharacterized protein	(A)GMPVHSVEDLPGAPFGDA(G)	2.57×10^7
	Glycinin G1	(A)VSIIDTNSLENQLDQ(M)	4.56×10^7
		(S)IIDTNSLENQLDQMPR(R)	2.07×10^7
		(G)ANSLLNALPEEVIQ(H)	2.25×10^7
	Hydrolase_4 domain-containing protein	(A)AAEGGGFSDPAPAPPRLAIPEV(P)	1.45×10^7
	DNA-directed RNA polymerase (Fragment)	(L)FDIYRVMRPGEPPTMDSAEAMFNA(L)	1.48×10^7
	Heterokaryon incompatibility protein	(L)GGLVQPIQMSKSARADGGDVSAQLANLDLS(A)	1.64×10^7
	Uncharacterized protein	(Q)HGLGLEVIELGNCMVDGFYLSSR(S)	4.81×10^7
	Phosphatidylinositol-specific phospholipase C	(H)DNDIATALSNLGIFTFSEQ(F)	1.15×10^7
	Uncharacterized protein	(P)LQRIGVGLVFSILAMVSAALI(E)	2.57×10^7
	Uncharacterized protein	(K)HKYVVPIPVIAMATGESG(E)	5.83×10^7
	C-x8-C-x5-C-x3-H type zinc finger protein	(N)TAVDRTLADFGRGFGRG(Q)	2.12×10^7
	PRONE domain-containing protein	(S)PQVPKSGLS(D)	1.34×10^7
	PH domain-containing protein	(P)PSISSQSRASSDSSSK(E)	9.54×10^6
	GMC_OxRdtase_N domain-containing protein	(N)AGFYSRADADFFARS(G)	2.08×10^7
PH	Vicilin	(E)ITPEKNPQLQDLDIFVN(S)	3.14×10^7
		(E)KNPQLQDLDIFVN(S)	5.96×10^7
	Vicilin 47k	(F)EITPEKNQQLQDLDIFVN(S)	2.26×10^7
		(E)KNQQLQDLDIFVN(S)	7.09×10^7
		(K)NQQLQDLDIFVN(S)	4.01×10^7
	Legumin A2	(N)ALEPDNRIE(S)	1.53×10^7
		(S)SVINNLPLDVVA(A)	4.96×10^7

Mannonate dehydratase	(T)GATNIVSSLHQVPIGRAWT(E)	3.28×10^{7}
LysR family transcriptional regulator	(K)HLFILGGLGWGGLPASVVKDDL(A)	1.04×10^{8}
Aldehyde dehydrogenase	(T)GATAQWAAINCGLGADILREAA(A)	1.70×10^{7}
Leucine-rich repeat receptor-like protein	(F)GIDLSNNLLHGEIPRGLFGLAGLE(Y)	3.11×10^{7}
AsmA family protein	(S)GGLSFDRKAAKTTASGGLTLSKADA(G)	2.73×10^{7}
	(I)LFGQAGLDPLPVDVGANGRL(T)	1.80×10^{7}
TP-binding protein	(L)DRMFCGIIDRDGGAPGTDRIF(P)	3.23×10^{7}
Putative aromatic aminotransferase protein	(A)TFIQAAVPRIIT(Q)	3.03×10^{7}
Argonaute 2	(Q)WPCLQVGNPQRPNYLPMEVCKIVEG(Q)	2.95×10^{7}
ABC transporter substrate-binding protein	(G)WAGAAFGFEESPELKALVDAGKLPPVE(K)	6.51×10^{7}
ABC transporter substrate-binding protein	(S)GGGTWEAAQKKAFFDPFTRDTGIKVV(L)	5.85×10^{7}
Mannonate dehydratase	(I)RGGKLSFMETFPDEGDMDMVRS(V)	5.80×10^{7}
LysR family transcriptional regulator	(K)HLFILGGLGWGGLPASVVKDDL(A)	2.92×10^{7}
Clink	(F)SQLPEELKEKIMNEHLKEI(K)	2.79×10^{7}
Peptidoglycan-associated protein	(K)KPPNSAGDLGLGTGAGGAATPGSAQDFTVNV(G)	6.60×10^{7}
Sporulation protein	(P)ITPAPQQVAAVSPRPAPVFA(P)	1.82×10^{7}
Dioxygenase RAMOSUS5	(P)KPVPAPAPIPTTDVVIPGRILQPVQPFI(D)	1.36×10^{7}
Putative DNA modification/repair radical SAM protein	(L)NIELPTDSGITRF(A)	2.14×10^{7}
Hydantoinase/oxoprolinase family protein	(Y)EGDVLVSTSIGGCNQISDVISKPIQLAK(S)	3.06×10^{7}
Aspartate/tyrosine/aromatic aminotransferase	(F)IDLAYQGLGDGLEQDAAPARM(V)	1.59×10^{7}
DNA replication licensing factor MCM3	(G)THLRGDINMMMVGDPSVAKS(Q)	2.79×10^{7}
L-threonine 3-dehydrogenase	(V)PMVVGHEFSGEIAEIGSAVTRY(H)	4.36×10^{6}

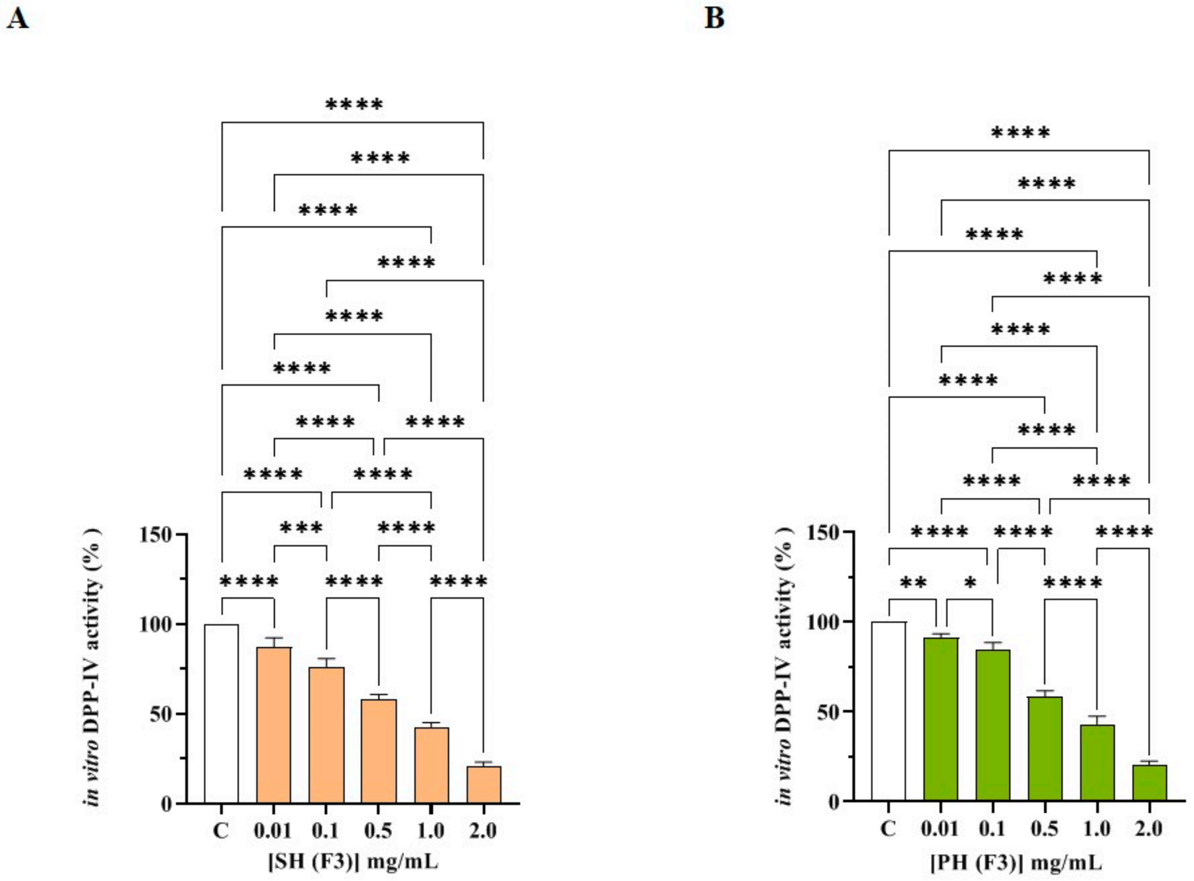

Figure S1: Evaluation of the *in vitro* inhibitory effects of SH (F3) (A) and PH (F3) (B) hydrolysates on human recombinant DPP-IV. Bars represent the average ± s.d. of three independent experiments in duplicates. **** $p < 0.0001$, *** $p < 0.001$, ** $p < 0.01$, * $p < 0.05$ versus control (C) sample (Activity).

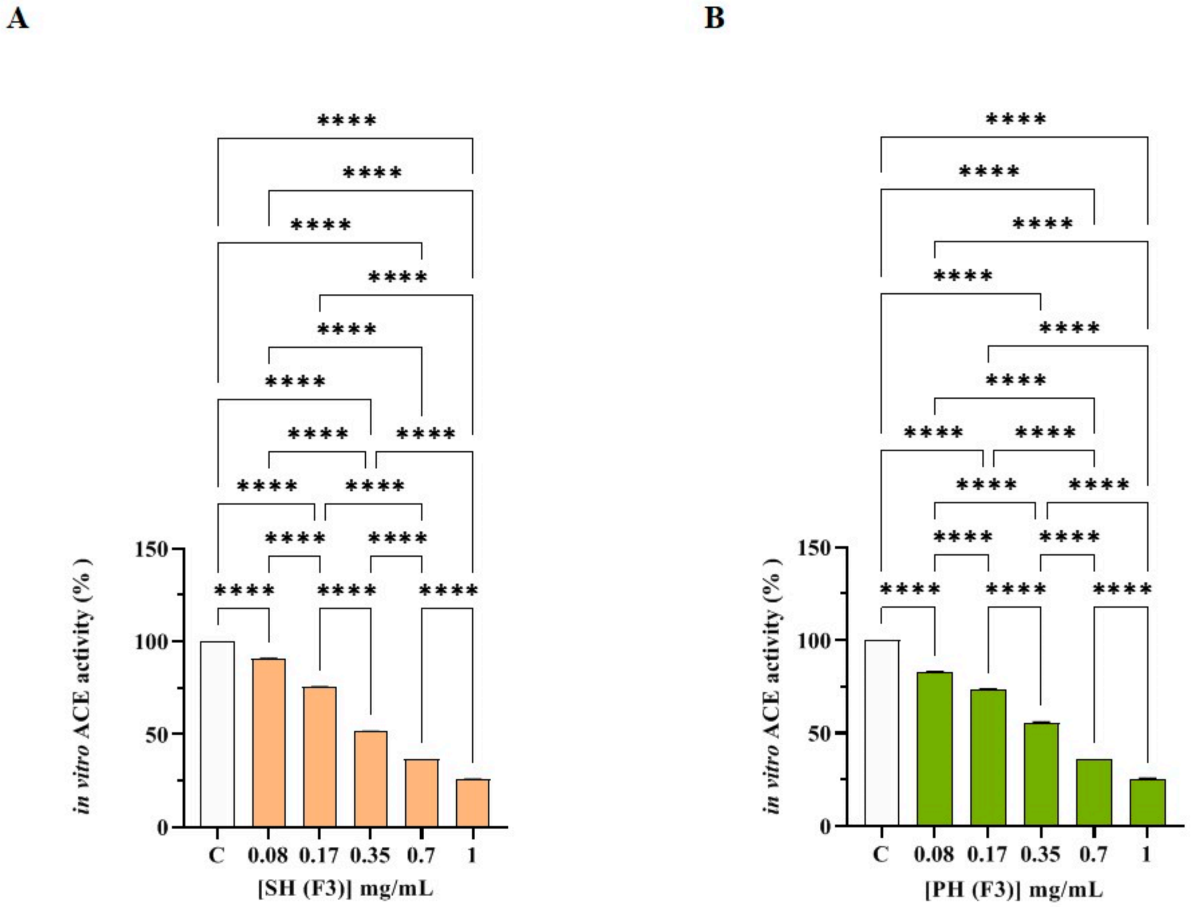

Figure S2: Evaluation of the *in vitro* inhibitory effects of SH (F3) (**A**) and PH (F3) (**B**) hydrolysates on ACE. Bars represent the means ± sd of three independent experiments in duplicate. **** $p < 0.0001$ versus Control sample (C).

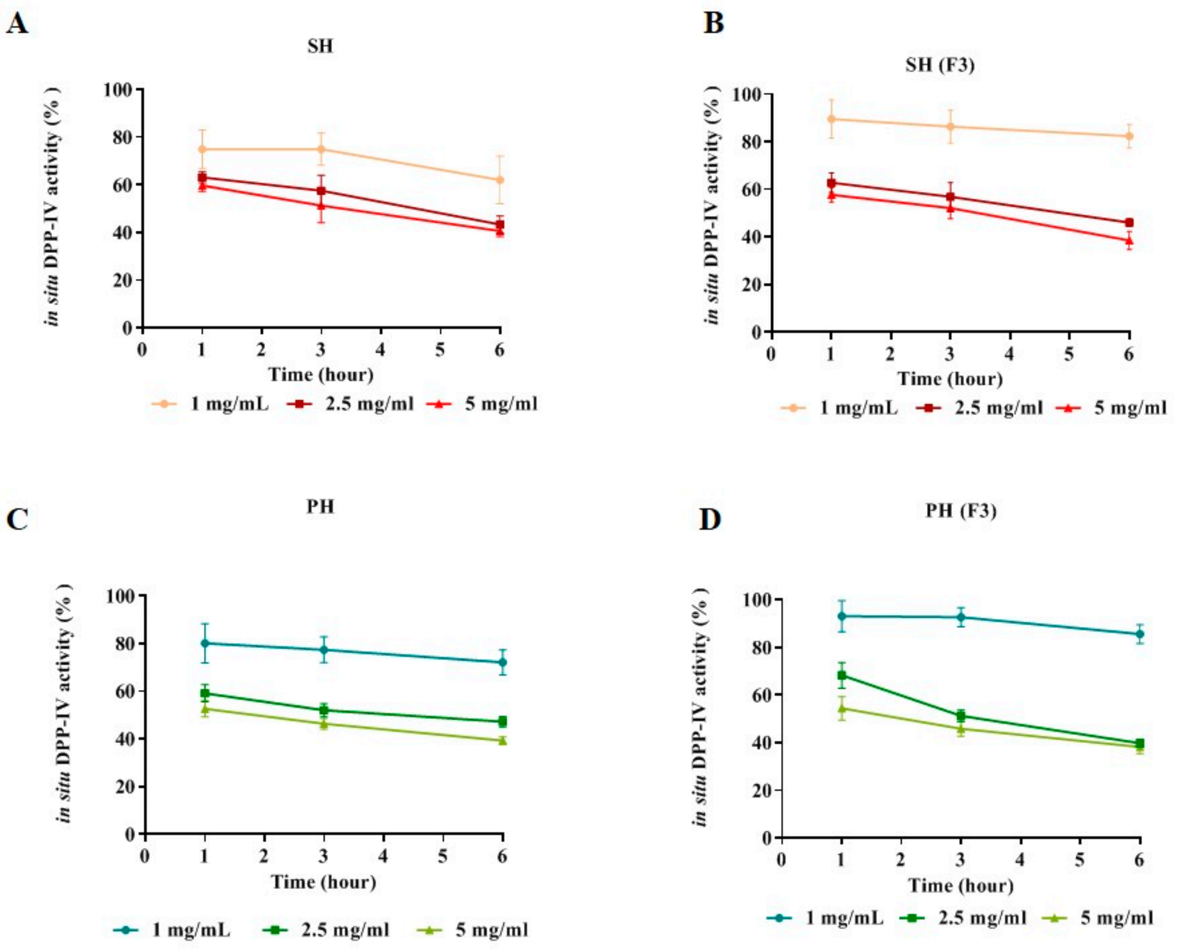

Figure S3: The kinetics of the inhibition of cellular DPP-IV activity after incubating Caco-2 cells with the SH (**A**), SH (F3) (**B**), PH (**C**) and PH (F3) (**D**) hydrolysates for 1, 3 and 6 hours at different concentrations. The data are represented as the means ± s.d. of four independent experiments, performed in triplicate.

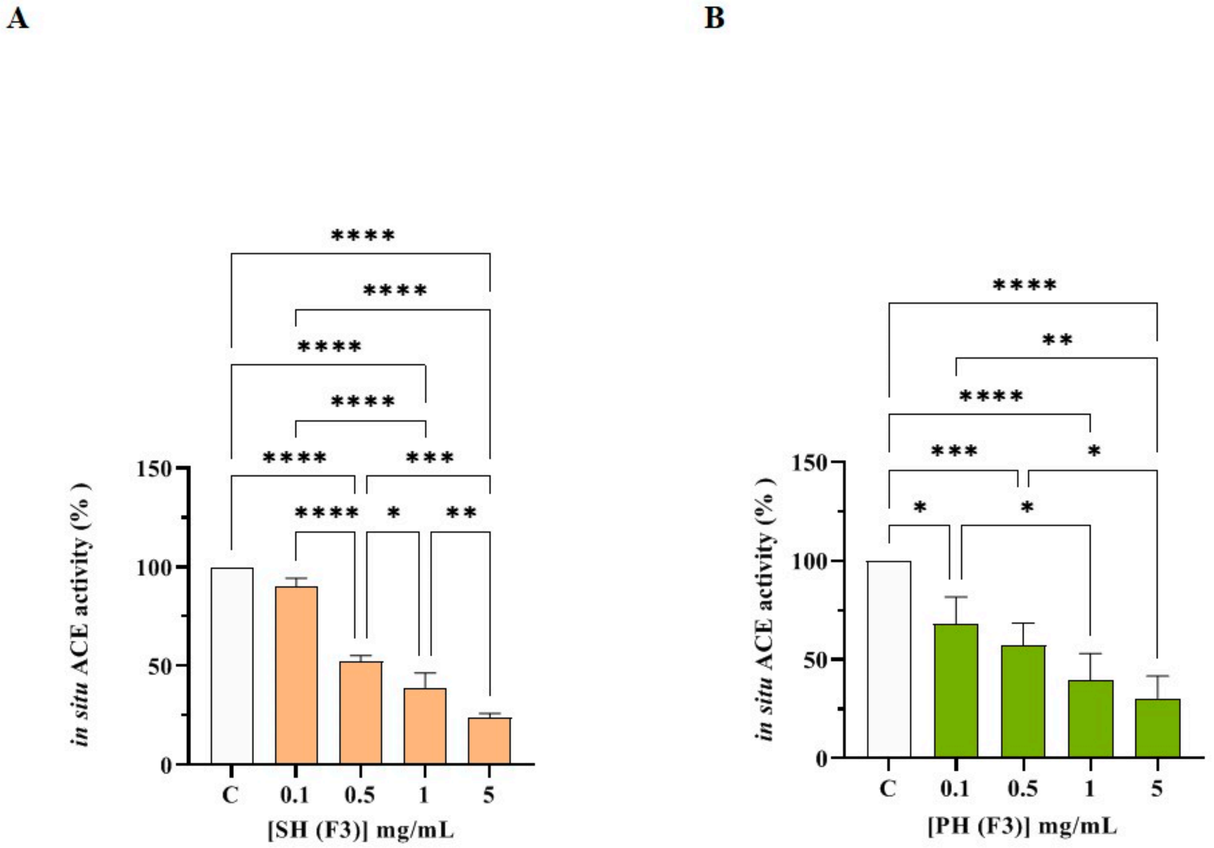

Figure S4: Evaluation of the inhibitory effects of SH (F3) (**A**) and PH (F3) (**B**) hydrolysates on ACE expressed on Caco-2 cells membranes. Bars represent the SD of three independent experiments in triplicate. **** $p < 0.0001$, *** $p < 0.001$, ** $p < 0.01$, * $p < 0.05$ versus Control sample (C), non-significant (ns) is not shown.
